# The Prescription of Oral Mucosal Mesenchymal Stem Cells post-Traumatic Brain Injury Improved the Kidney and Heart Inflammation and Oxidative Stress

**DOI:** 10.1155/2022/8235961

**Published:** 2022-11-10

**Authors:** Maryam Radavi-Asgar, Nazanin Sabet, Mohammad Khaksari, Elham Jafari, Zahra Soltani, Fatemeh Dehghanian

**Affiliations:** ^1^Neuroscience Research Center, Institute of Neuropharmacology, Kerman University of Medical Sciences, Kerman, Iran; ^2^Physiology Research Center, Institute of Neuropharmacology, Kerman University of Medical Sciences, Kerman, Iran; ^3^Endocrinology and Metabolism Research Center, Institute of Basic and Clinical Physiology Sciences, Kerman University of Medical Sciences, Kerman, Iran; ^4^Department of Physiology and Pharmacology, Afzalipour School of Medicine, Kerman University of Medical Sciences, Kerman, Iran; ^5^Pathology and Stem Cells Research Center, Department of Pathology, Afzalipour School of Medicine, Kerman University of Medical Sciences, Kerman, Iran; ^6^Research Center of Tropical and Infectious Diseases, Kerman University of Medical Sciences, Kerman, Iran; ^7^Non Communicable Diseases Research Center, Bam University of Medical Sciences, Bam, Iran; ^8^Bam University of Medical Sciences, Bam, Kerman, Iran

## Abstract

**Background:**

In the last years, mesenchymal stem cells (MSCs) have been considered as a useful strategy to treat many diseases such as traumatic brain injury (TBI). The production of inflammatory agents by TBI elicits an inflammatory response directed to other systems of body, such as the heart and the kidneys. In this study, the efficacy of oral mucosal mesenchymal stem cells (OMSCs) prescription after TBI in inflammation and oxidative stress of the heart and kidneys was evaluated.

**Methods:**

Twenty-four male rats were located in groups as follows: sham, TBI, vehicle (Veh), and stem cell (SC). OMSCs were injected intravenously 1 and 24 hours after TBI. Inflammatory, oxidative stress, and histopathological outcomes of the heart and kidney tissues were investigated 48 hours after TBI.

**Results:**

TBI caused an increase in the level of interleukin-1*β* (IL-1*β*), interleukin-6 (IL-6), malondialdehyde (MDA), and carbonyl protein (PC) of the heart and kidney compared to the sham group. Superoxide dismutase (SOD), total antioxidant capacity (TAC), catalase (CAT), and interleukin-10 (IL-10) of the heart and kidney decreased after TBI. The use of OMSCs after TBI reduced the changes of these factors in both the heart and the kidney.

**Conclusion:**

Application of OMSCs after TBI can decrease inflammation and oxidative stress of the heart and kidney tissues leading to the reduction of damage. Therefore, this method can be evaluated in the TBI patients in future studies.

## 1. Introduction

Traumatic brain injury (TBI) is one of the common and growing neurological disorders worldwide [1]. Population-based studies have shown that more than 50–60 million people worldwide are affected by a new TBI annually [2]. Road traffic accidents, falls from height, injuries caused by intense exercise, and assault are the main causes of TBI [3].

TBI can include two injuries: early and late that occurs after the early injury. Early injury is created through the pressure of an external object which results in a wave of vascular, cellular, metabolic, and neurochemical disturbances that cause late damage [1]. Some of mechanisms of late brain injury include increased reactive oxygen species (ROS), increased energy expenditure, damage to blood-brain barrier (BBB), inflammation, membrane lipid peroxidation, advanced neuronal demolition, apoptosis, and liberation of excitatory amino acids [4, 5].

The importance of inflammatory and oxidant factors in the development of systemic damage after TBI has been considered [6]. Overproduction of ROS and weakness of antioxidant protection system perform a significant role in creation of oxidative stress after TBI [7]. Cardiovascular dysfunction and increased mortality have been reported to be prevalent in people suffering from severe brain injury [8–10]. Pathological factors include oxidative stress release cytokines from cardiomyocytes [11]. Immune cells and inflammatory factors generated by brain injury lead to collagen accumulation, propagation of cardiac fibroblasts, and loss of cardiomyocytes [11]. In addition, TBI is related to the progression of acute renal failure (ARF). Systemic inflammation following trauma and the onset of cytokine cascade due to brain injury may impair blood flow to body organs and excretory function [12, 13].

The goal of treatment in intracranial trauma is to prevent the development of secondary injuries [14]. Despite the importance of this problem, no treatment has been explicitly approved for TBI [15]. Perhaps, one reason for the lack of treatment is the heterogeneous and systemic pathological processes of TBI that have been disregarded in TBI research. The basic suggestions for TBI treatment investigations include the choice of therapies that have numerous objectives instead of a solitary target [16], thus targeting TBI multiple pathological and systemic processes [17].

The application of stem cells (SCs) has considered as a useful strategy to treat neurological diseases such as TBI [17]. Given, the positive effects of mesenchymal stem cells (MSCs) such as the high ability in inflammation repression, differentiation, the regulation of immune system, and the rehabilitation of neurological disorders such as TBI have been considered [18, 19]. It has been shown that mesenchymal cells in TBI selectively transfer to the damaged site, differentiate to neurons, and improve neurologic results [20]. The possible mechanism of the mentioned processes at the first stage is the liberation of chemokines, growth factors [21], and adhesive molecules, which connect these cells to the damaged endothelium [22]. Since adhesive and inflammatory factors and the activating natural T cell increase infiltration of leukocytes into the brain, using MSCs reduce inflammatory leukocytes in the site of injury through downregulating of these factors [5]. The use of bone marrow-derived MSCs (BM-MSCs) in brain damage decreases the microglia lysosomes which are more basic agents in increasing tumor necrosis factor (TNF-*α*) and interleukin-6 (IL-6) in the damaged area [23, 24]. Among MSCs, oral mesenchymal stem cells (OMSCs) are suggested to be used in the study of TBI treatment, because they are described as cells with neural crest origin [25], and they have neuroprotective effect in the laboratory [26] and in living organisms [25]. Several studies have shown that the transplantation of embryonic cardiomyocytes and BM-MSCs can regenerate myocardial cells from scar or infarcted tissues and strengthen the work of the heart in animal models [27]. Also, administration of MSCs following myocardial ischemia reduces oxidative stress [28].

In the experimental study by Morigi et al. (2004)[29], it was shown that administration of MSCs in mice with ARF repaired renal structure and improved renal function due to differentiation of MSCs into tubular epithelium [29]. Also, in a study, administration of MSCs in rats suffering from ARF decreased serum creatinine and the expression of inflammatory cytokines, and on the other hand, improved renal function and the expression of anti-inflammatory cytokines [30].

Due to the systemic damage that occurs in TBI following systemic inflammation and also regarding the beneficial effect that MSCs have on TBI, this study intended to assess changes in inflammatory, oxidative stress, and histopathological consequences of the heart and the kidney after administration of OMSCs in rat model with diffuse TBI.

## 2. Material and Methods

### 2.1. Animals

In this experiment, adult male Wistar rats weighing 200-250 g were provided from Laboratory Animal Unit of Kerman University of Medical Sciences (KMUS). They were housed in cages under a 12 h/12 h light-dark cycle, at room temperature (22–25^*ο*^C) with free access to food and water ad libitum. Animal care committee of Kerman University of Medical Sciences, Kerman, Iran, approved the experiments (ethics code: IR.KMU.AH.REC.1400.065 and ID: 99000804).

### 2.2. Study Groups

**Figure 1:**
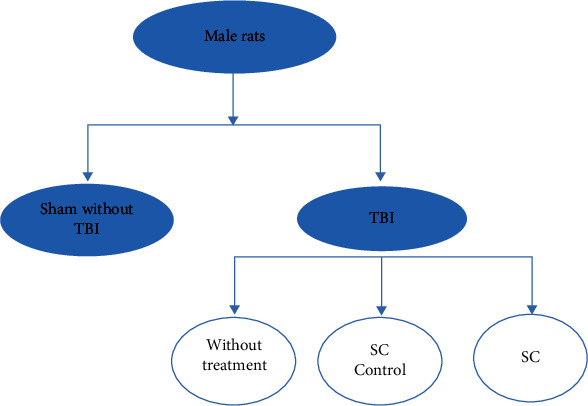
The diagram of the research groups.

The study groups (*n* = 6 in each group) were as follows: (1) sham (male rats were anesthetized and then, all steps of TBI induction were carried out except the weight falling upon their head) [31, 32]; (2) TBI (male rats with diffuse TBI); (3) SC control or vehicle (Veh) (male rats that received the same amount of intravenous buffer saline phosphate solution as the SC injection (100 *μ*l) at 1 and 24 hours after TBI); and (4) SC (male rats that received 2 × 10^6^ SC per 100 *μ*l PBS intravenously at 1 and 24 hours after TBI). The diagram of study groups is shown in Figure 1.

### 2.3. Measurement of Inflammatory and Anti-Inflammatory Cytokines in the Kidney and Heart

The amounts of inflammatory (IL-1*β* and IL-6) and anti-inflammatory (IL-10) agents in homogeneous solution of the heart and kidney tissues were measured using the relevant kits (KPG-MIL-1*β*, KPG-MIL-6, and KPG-MIL-10, respectively, Pars Gene Karmania Kit, Iran) and the ELISA method. Eventually, the absorption was record at 450 nm. Following obtaining the standard curve, the concentration of samples was evaluated and expressed using the standard curve in picogram per milligram of protein [33, 34].

### 2.4. Assessment of Oxidant and Antioxidant Indices

#### 2.4.1. Amounts of Malondialdehyde Levels of the Kidney and the Heart

About 48 h after TBI induction, the level of malondialdehyde (MDA) as an index of oxidative stress was assessed by thiobarbituric acid (TBA) method. Briefly, to the homogenized tissue of the heart and the kidney, a reaction mixture containing TBA, sodium dodecyl sulfate (SDS), acetic acid, and distilled water was appended. The resulting mixture was heated, and following cooling, it was centrifuged to prepare a smooth solution. Then, the absorption of supernatant fluid was read at 532 nm. The data were presented in units of nanomoles per milligram of protein and calculated through a standard curve [34].

#### 2.4.2. Kidney and Heart Protein Carbonyl Content

Protein carbonyl (PC) is a marker of protein oxidation and indicates oxidative activity which was evaluated via 2, 4-dinitrophenylhydrazine (DNPH) method. The homogeneous solution of the kidney or heart tissue was filtered through 8 layers of muslin to remove the cytoskeleton. Then, the filtered solution containing protein was mixed with DNPH dissolved in solution of hydrochloric acid. The samples were incubated with shaking at room temperature, and then, they were deposited with TCA and centrifuged. After rinsing, the samples were placed in guanidine and then centrifuged to remove insoluble materials. The difference in absorption between NDPH and HCL samples was read at 366 nm and was expressed as nanomole per milligram of protein [35].

#### 2.4.3. Kidney and Heart Total Antioxidant Capacity Content

Evaluation of total antioxidant capacity (TAC) was performed using the ferric reducing antioxidant power (FRAP) method. The homogenized tissues of the heart and the kidney were centrifuged. The precipitate was removed, and the supernatant was obtained and then it was diluted 5 times with distilled water. Then, from the diluted homogenized samples, distilled water (as a control) and iron sulfate were appended to the tubes containing FRAP and then were heated at 37°C. The adsorption of the samples was read at 593 nm and expressed as nanomole per milligram of tissue [36].

#### 2.4.4. Kidney and Heart Superoxide Dismutase Content

For evaluation of superoxide dismutase (SOD) enzyme, EDTA in sodium cyanide and nitro blue tetrazolium (NBT) were added to a certain volume of homogenized solutions of heart or kidney tissues and after mixing, it was placed at 37°C. Then, riboflavin and methionine were added to the samples and the volume was increased to 3 ml and placed at environment temperature and light for 10 minutes. Then, the absorption of samples and control (distilled water) was read at 560 nm for 5 minutes. Enzyme activity was reported based on units per mg of protein [37].

#### 2.4.5. The Amount of Catalase Activity of the Kidney and the Heart

To measure the activity of catalase (CAT) enzyme, the homogenized samples of the heart or the kidney tissues were centrifuged. The supernatant solution was applied for measurement. To a certain volume of this fluid, potassium phosphate buffer and oxygenated water were appended, and the absorption rate was determined at 240 nm per minute (every 15 seconds) and the absorption reduction rate per minute was calculated. The amount of enzyme activity was evaluated in units of tissue protein. One unit of activity of the enzyme catalase is the amount of enzyme that breaks down one millimole of oxygenated water per minute [38].

### 2.5. Evaluation of Histopathological Outcome

#### 2.5.1. Histopathological Evaluation of the Kidney and the Heart

Histopathological findings of kidney and heart were assessed 48 hours following TBI. Left kidney and left ventricle of the heart fixed in formalin were embedded in paraffin and cut into five *μ*m thickness sections. All slides were stained with hematoxylin and eosin. Blind pathologist reviewed the kind and severity of lesions. These results in kidney were in terms of inflammation, necrosis, interstitial edema, loss of brush border, vascular destruction, interstitial hemorrhage, and renal capsule dilatation. The alterations were scored as follows:

0 = no alteration, 1 = very mild alteration, 2 = mild alteration, 3 = moderate alteration, 4 = marked alteration, and 5 = very marked alteration [39].

Histopathological findings of the heart were in terms of necrosis, myofibrillar degeneration, vascular congestion, hemorrhage, leukocyte infiltration, and interstitial edema were scored as follows [40–42]:

0 = no damage, 1 = minimal damage , 2 = mild damage (myofibrillar degeneration of several small areas with a mild grade of inflammation), 3 = moderate damage (moderate degree of myofibrillar deterioration or spread inflammation), and 4 = intense damage (necrosis along with spread inflammation).

#### 2.5.2. Statistical Analysis

The results have been presented as mean ± SEM and the evaluation has been done by one-way ANOVA and post hoc Tukey's test using SPSS software version 20. *P* value less than 0.05 (*P* < 0.05) was set as statistically significant.

## 3. Results

### 3.1. Inflammatory and Anti-Inflammatory Indicators of the Kidney and the Heart following OMSCs Administration after TBI

#### 3.1.1. Evaluation of IL-1*β* Level

**Figure 2:**
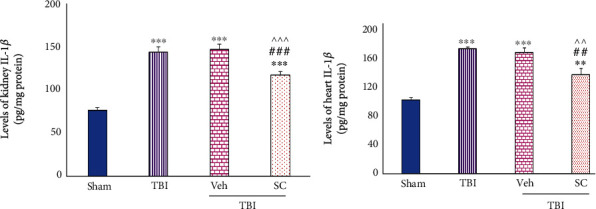
Evaluation of interleukin-1*β* (IL-1*β*) levels in OMSCs administration after TBI. (a) IL-1*β* level in the kidney. (b) IL-1*β* level in the heart. ^∗∗∗^*P* < 0.001 compared to sham. ^∗∗^*P* < 0.001 compared to sham. ^###^*P* < 0.001 compared with TBI. ^##^*P* < 0.01 compared to TBI. ^^^^^*P* < 0.001 compared to Veh. ^^^^*P* < 0.01 compared with Veh.

IL-1*β* levels in TBI, Veh, and SC groups were higher than the sham in kidney (*P* < 0.001). This increase was also seen following TBI in the heart after TBI (*P* < 0.001, *P* < 0.001, and *P* < 0.01, respectively). These levels were similar between TBI and Veh, but less increase in IL-1*β* levels following OMSCs administration after TBI was observed in both the kidney (*P* < 0.001) and the heart (*P* < 0.01) compared with TBI and Veh (Figure 2).

#### 3.1.2. Evaluation of Level of IL-6

**Figure 3:**
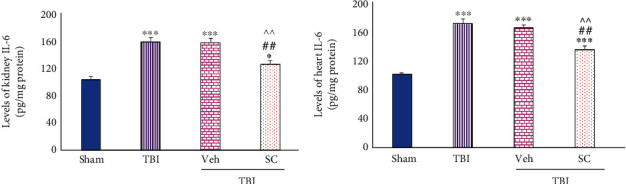
Evaluation of interleukin-6 (IL-6) levels in OMSCs administration after TBI (a) IL-6 level in the kidney. (b) IL-6 level in the heart. ^∗∗∗^*P* < 0.001 compared to sham. ^∗^*P* < 0.05 compared with sham. ^##^*P* < 0.01 compared with TBI. ^^^^*P* < 0.01 compared to Veh.

IL-6 level of kidney tissue increased in TBI, Veh, and SC in compared to sham (*P* < 0.001, *P* < 0.001, and *P* < 0.05, respectively). This increase was also seen following TBI in the heart (*P* < 0.001). These levels were similar between TBI and Veh, but less increase in IL-1*β* levels following OMSCs administration was observed in both kidney and heart tissues following TBI in comparison with TBI and Veh (*P* < 0.01), (Figure 3).

#### 3.1.3. Evaluation of IL-10 Level

**Figure 4:**
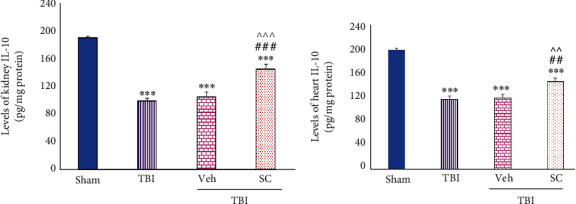
Evaluation of interleukin-10 (IL-10) levels in OMSCs administration after TBI. (a) IL-10 level of kidney. (b) IL-10 level of heart. ^∗∗∗^*P* < 0.001 compared to sham. ^###^*P* < 0.001 compared to TBI. ^##^*P* < 0.01 compared to the TBI group. ^^^^^*P* < 0.001 compared to the Veh group. ^^^^*P* < 0.01 compared to the Veh group.

IL-10 levels in both tissues were lower than sham following TBI (*P* < 0.001). After OMSC use following TBI, less changes were observed in the IL-10 level in both the kidney (*P* < 0.001) and the heart (*P* < 0.01) in comparison with TBI and Veh, (Figure 4).

## 4. Oxidant and Antioxidant Indicators of the Kidney and the Heart in OMSCs Administration after TBI

### 4.1. Evaluation of Oxidative Stress Indicators

#### 4.1.1. Evaluation of MDA Level

**Figure 5:**
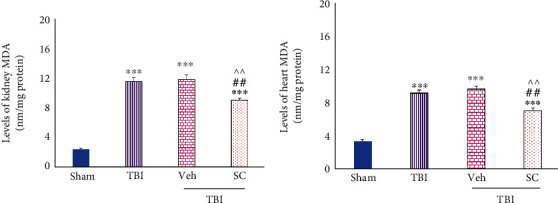
Evaluation of malondialdehyde (MDA) levels in OMSCs administration after TBI. (a) MDA level in the kidney. (b) MDA level in the heart. ^∗∗∗^*P* < 0.001 compared to the sham group. ^##^*P* < 0.01 compared to TBI. ^^^^*P* < 0.01 compared to the Veh group.

MDA levels in both tissues were higher than sham after TBI (*P* < 0.001). Following the administration of OMSCs, the level of MDA of kidney and heart tissues increased slightly less in comparison with TBI and Veh (*P* < 0.01), (Figure 5).

#### 4.1.2. Evaluation of PC Level

**Figure 6:**
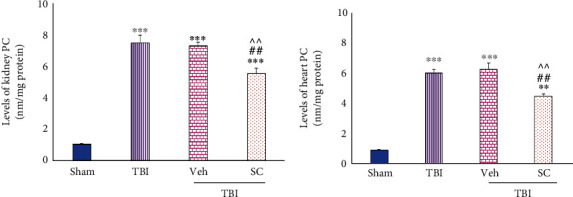
Evaluation of protein carbonyl (PC) levels in OMSCs administration after TBI. (a) PC level in the kidney. (b) PC level in the heart. ^∗∗∗^*P* < 0.001 compared with sham. ^∗∗^*P* < 0.01 compared with sham. ^##^*P* < 0.01 compared with the TBI group. ^^^^*P* < 0.01 compared with the Veh group.

Increased level of PC of kidney tissue after TBI was observed in all groups (*P* < 0.001). Also, in the heart tissue, TBI, Veh, and SC were higher than sham (*P* < 0.001, *P* < 0.001, and *P* < 0.01, respectively). After using OMSCs following TBI, a smaller increase in PC level was seen in both the kidney and the heart compared with TBI and Veh (*P* < 0.01), (Figure 6).

#### 4.1.3. Evaluation of TAC Level

**Figure 7:**
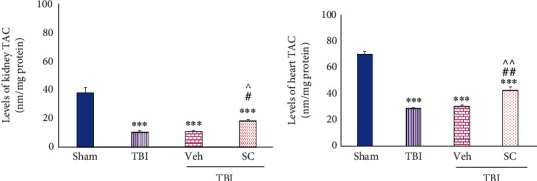
Evaluation of total antioxidant capacity (TAC) levels in OMSCs administration after TBI. (a) TAC levels in the kidney. (b) TAC levels in the heart. ^∗∗∗^*P* < 0.001 comparison with sham. ^##^*P* < 0.01 comparison with TBI. ^#^*P* < 0.05 compared with TBI. ^^^^*P* < 0.01 comparison with Veh. ^^^*P* < 0.05 compared with Veh.

Amount of TAC in both tissues decreased after TBI in all groups comparison with sham (*P* < 0.001) and the rate of reduction did not differ between TBI and Veh. Administration of OMSCs after TBI caused less reduction in level of TAC in both the kidney (*P* < 0.05) and the heart (*P* < 0.01) in comparison with TBI and Veh, (Figure 7).

#### 4.1.4. Evaluation of SOD Level

**Figure 8:**
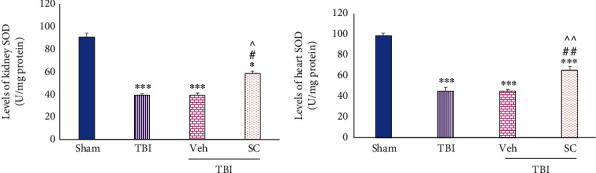
Evaluation of superoxide dismutase (SOD) levels in OMSCs administration after TBI. (a) SOD levels of kidney. (b) SOD levels of heart. ^∗∗∗^*P* < 0.001 comparison with sham. ^∗^*P* < 0.05 compared with sham. ^##^*P* < 0.01 comparison with TBI. ^#^*P* < 0.05 compared with TBI. ^^^^*P* < 0.01 comparison with Veh. ^^^*P* < 0.05 comparison with Veh.

After TBI, the SOD levels of TBI, Veh, and SC groups in the kidney decreased (*P* < 0.001, *P* < 0.001, and *P* < 0.05, respectively). Also, this level decreased in heart in all groups in comparison with sham (*P* < 0.001). This reduction rate was less in SC compared with TBI and Veh in both the kidney (*P* < 0.05) and the heart *P* < 0.01), (Figure 8).

#### 4.1.5. Evaluation of CAT Level

**Figure 9:**
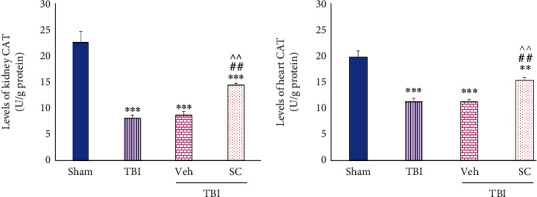
Evaluation of catalase (CAT) levels in OMSCs administration after TBI. (a) CAT level in the kidney. (b) CAT level in the heart. ^∗∗∗^*P* < 0.001 comparison with sham. ^∗∗^*P* < 0.01 compared with sham. ^##^*P* < 0.01 comparison with TBI. ^^^^*P* < 0.01 comparison with Veh.

Decrease in the level of CAT of kidney was observed in all groups (*P* < 0.001) and in heart tissue, it was seen in TBI, Veh, and SC (*P* < 0.001, *P* < 0.001, and *P* < 0.01, respectively) in comparison with sham. Following OMSCs administration after TBI, the changes of level of CAT were less in kidney and heart tissues compared with TBI and Veh (*P* < 0.01), (Figure 9).

### 4.2. Histopathological Outcome of the Kidney and the Heart in OMSCs Administration after TBI

**Figure 10:**
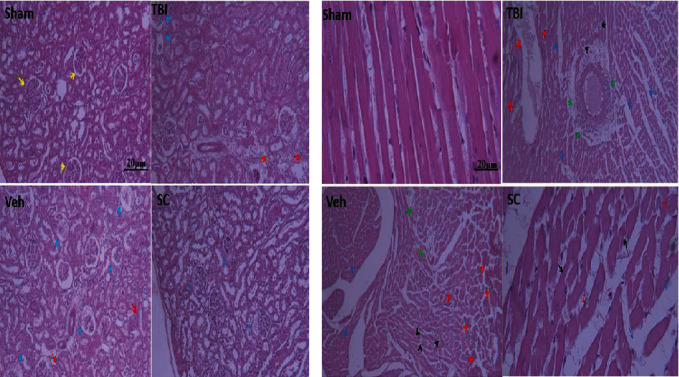
Histopathological changes of kidney and heart tissues in OMSCs administration after TBI. Photomicrographs of hematoxylin and eosin–stained sections of the kidney and heart tissues of rat with X100 magnification, scale bar: 20 microns. (a) Histopathological changes of kidney tissue are shown as follows: renal cortex shows preserved architecture and normal glomeruli (yellowish arrows). Renal cortex shows shrinkage glomeruli with hemorrhage in renal corpuscle (bluish arrows) and extensive tubular cells necrosis with brush border loss and desquamation along with cast formation and focally chronic inflammatory cells infiltration (red arrows). (b) Histopathological changes of heart tissue are shown as follows: edema (bluish arrows), heart tissue with necrotic and destructed cardiomyocytes (red arrows), vascular congestion and chronic inflammatory cells infiltration (green arrows), and hyper eosinophilic bundles (black arrows).

In kidney tissue, the rate of glomerular hemorrhage, necrosis, inflammatory cell infiltration, loss of examination edges, and cast formation are enhanced in TBI and Veh compared to sham. No difference was seen in this increase between TBI and Veh. Administration of OMSCs led to less changes in comparison with TBI and Veh. In heart tissue, the level of edema, necrosis, and destruction of cardiomyocytes, vascular congestion, infiltration of chronic inflammatory cells, and hypereosinophilic bundles in the heart tissue increased following TBI compared with sham. Rate of increase was similar between the TBI and the Veh. Following the administration of OMSCs, this increase was lower comparison with TBI and Veh, (Figure 10).

### 4.3. The Total Score of Heart and Kidney Damage in OMSCs Administration after TBI

**Figure 11:**
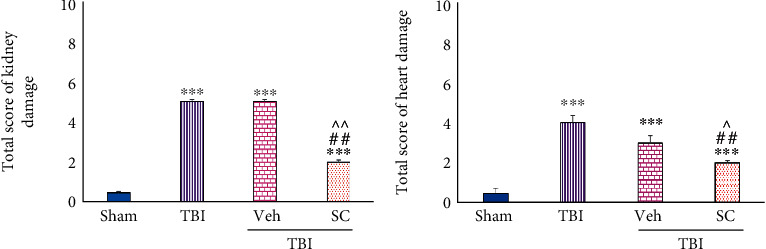
Evaluation of total score of damage in OMSCs administration after TBI. (a) Total score of kidney damage. (b) Total score of heart damage. ^∗∗∗^*P* < 0.001 comparison with sham. ^##^*P* < 0.01 compared with TBI. ^^^^*P* < 0.01 compared with Veh. ^^^*P* < 0.05 comparison with Veh.

The total score increased in both heart and kidney tissues at all groups in comparison with sham (*P* < 0.001), and increase was similar in the heart and kidney tissues between TBI and Veh. Administration of OMSCs resulted in less increase in the score of damage in the kidney (*P* < 0.01) and the heart (*P* < 0.01 and *P* < 0.05, respectively) compared to TBI and Veh, (Figure 11).

## 5. Discussion

In this research, for the first time, the effectiveness of OMSC use following diffuse TBI on inflammatory factors, oxidative stress, and histopathological outcome of the kidney and the heart in male rats was investigated.

The findings of the present study showed that (1) Following TBI, levels of inflammatory cytokines in the heart and kidney tissues increased while the amount of anti-inflammatory cytokine of the heart and the kidney decreased. Administration of OMSCs following TBI, the rate of these changes reduced. (2) The level of oxidative factors such as MDA and PC increased following TBI in the heart and kidney tissues, but the level of antioxidative factors such as TAC, SOD, and CAT of the heart and kidney tissues decreased. These changes were less prominent with administration of OMSCs. Administration of OMSCs following TBI also improved the histopathological outcome of the heart and kidney tissues.

It has been reported that secondary damage in TBI can cause systemic inflammation via increasing the generation of inflammatory agents associated with decreasing anti-inflammatory agents which this systemic inflammation can damage other tissues such as the kidney and the heart [6]. MSCs have been suggested as anti-inflammatory agents due to their ability to interact rapidly with the environment and being stimulated [43]. MSCs are often used in inflammatory disorders to modulate the immune function, and repression of immune system can prevent the production of ROS by the enzymes that are part of the inflammatory response [44].

In current research, inflammatory factors of the heart and kidney increased following TBI. In other tissues, similar results have been reported. Lackner et al. reported that IL-1*β* in mice's heart tissue increased 24 h after TBI [45]. Kalsotra et al. also observed an increase in IL-1*β* and IL-6 level in lung tissue of rat 24 hours after TBI [46]. Chen et al. showed elevation of IL-1*β* level at 1 and 6 hours, as well as on days 1 to 5 after TBI in intestinal tissue of rat, but they reported no change in levels of IL-6 [47]. Najafipour et al. did not observe change in level of these two inflammatory factors of heart rat 24 hours following TBI which may be due to differences in trauma severity and timing of post-Traumatic inflammatory factor evaluation [42]. This study showed a reduction in amount of IL-10 of the heart and the kidney after TBI. In study of Lackner et al., an increase in the heart tissue IL-10 was observed 24 hours after TBI in mice [45]. Probably the reason for the variability of the results is the type of animal, the trauma, and time of assessment of post-Traumatic factors.

In this study, after OMSC use following TBI, less changes in inflammatory factors were observed in both the heart and kidney tissues. Treatment with MSCs has been suggested that it helps to heal ARF in the animal model, and this reduces oxidative stress (decreased MDA and increased CAT and SOD), followed by decreased expression of nuclear factor kappa (NF-*κ*B), IL-1*β*, and IL-4 and IL-10 increased [48, 49]. Therapeutic effects of BM-MSC have been shown on the heart function in cardiac fibrosis, as BM-MSCs induce immunosuppression by increasing expression of IL-10 [50]. The exosomes secreted from MSCs play an important role in modulating myocardia recovery after myocardial infarction [51].

Among classic features of MSCs is their ability to transfer to injury and inflammation areas, as well as secrete cytokines and growth agents to augment regeneration, reduce inflammation, or change into various species of injured tissue [52]. MSCs decrease the rate of neutrophils that attach to vascular endothelial cells and this way limit the uptake of these cells into inflammatory sites [53]. In addition, MSCs have the potential to limit the secretion of proinflammatory chemokines via mast cells and restrict the migration and degranulation of these cells near chemotactic agents [53].

Overproduction of ROS and weakness of antioxidant system perform a prominent role in creation of oxidative stress after brain injury [9]. In the present study, following TBI, an increase in oxidative factors such as MDA and PC and a decrease in antioxidative factors of SOD, CAT, and TAC were observed in the heart and kidney tissues. To date, no study has been reported to investigate changes in oxidative factors in both the heart and kidney tissues following brain injury. In other tissues including liver, Amiresmaili et al. reported that oxidative factor of MDA increased and antioxidant enzymes such as SOD, 24 hours after TBI in rats, decreased [54]. de castro et al. in a research in rats observed a decrease in liver antioxidant enzymes (SOD and CAT) 24 hours following brain injury [55].

In current study, after OMSC use following TBI, less changes in oxidative stress factors were observed in both the heart and kidney tissues. In other study, Zhang et al. observed that BMSC use reduced MDA following heart damage [56]. Li et al. also reported that oxidative stress in lung injury improved after administration of multipotent mesenchymal cells [57].

The mechanism of oxidative stress reduction following MSCs can be mediated by the ability of these cells to secrete particles modulating immunity, such as exosomes which limit apoptosis, stimulate angiogenesis, and stem cell differentiation, inhibit oxidative reactions, and enhance extracellular matrix regeneration [58]. Maintaining mitochondrial function is essential in rescuing cells from oxidative stress. Mitochondrial function is impaired following brain injury [59]. Consumption of stem cells after brain injury increases mitochondrial transport capacity which begins with signaling of damaged cells, in which healthy mitochondria replace dysfunctional mitochondria [59].

MSCs perform immunosuppressive functions via reducing oxidative stress and increasing mitochondrial operation of macrophages and neutrophils. Similarly, enhancing the antioxidant properties of MSCs through increasing the expression of antioxidant genes or mitochondrial transport is likely to be involved in the management of MSCs-based TBI therapy and the improvement of outcomes [44].

In this study, pathological observations following TBI in the heart tissue included necrosis, interstitial edema, congestion and bleeding, leukocyte infiltration, and increased eosinophilic bandages. Also in kidney tissue, tubular necrosis, inflammation, interstitial edema, vascular degeneration, interstitial hemorrhage, renal capsular dilatation, and loss of brush border were seen after TBI. Tanaka and Okusa observed increasing of inflammation and fibrosis in the kidney after TBI [60]. Also, Zhao et al. reported that apoptosis, inflammation, and oxidants were significantly increased in heart 3 and 30 days following TBI [11]. One study showed that following TBI, histopathological changes such as edema, inflammation, and apoptosis appeared in other tissues, including the liver [54]. However, in the study of Najafipour et al. after TBI, no changes in histopathological outcome were observed 24 hours after injury which is probably due to the severity of trauma and the time of assessment of post-Traumatic histopathological outcome [42].

Tomita et al. reported that bone marrow cells (BMCs) in the human myocardium reduced ischemic heart damage by reducing the hypertrophy index, increasing angiogenesis, reducing scar area, and improving histopathological outcome [61]. Also, a study shown that administration of MSCs reduced inflammation and apoptosis following renal injury [30].

The finding indicated that using OMSCs after TBI decreased the damage to heart and kidney tissues which may have been done by modulating inflammation and reducing oxidative stress in these tissues. These results require further studies to confirm. Recent studies conducted on the transplantation of BMSCs, hematopoietic stem cells (HSCs), and MSCs show that SCs exert their beneficial effects through paracrine pathway [62, 63]. Determining the possible mechanism of OMSCs effect in reducing inflammation and oxidative stress will be the subject of future research.

## 6. Conclusion

The finding of the current study showed that using OMSCs may serve as an appropriate therapeutic agent in the treatment of TBI and reduce damage in the heart and kidney tissues probably part by the reduction of inflammation and oxidative stress. It is essential to conduct further investigation in order to determine the definite protective effect of OMSCs on patients suffering from TBI.

## Data Availability

The data of this study are available from the corresponding author upon reasonable request.

## Abbreviations

ARF: Acute renal failure
BBB: Blood-brain barrier
BMCs: Bone marrow cells
BM-MSCs: Bone marrow-derived MSCs
CAT: Catalase
DNPH: 2,4-Dinitrophenylhydrazine
EDTA: Ethylenediaminetetraacetic acid
FRAP: Ferric reducing antioxidant power
HSCs: Hematopoietic stem cells
IL-4: Interleukin-4
IL-6: Interleukin-6
IL-10: Interleukin-10
IL-1*β*: Interleukin-1*β*
MDA: Malondialdehyde
MIP-2: Macrophage inflammatory protein-2
MSCs: Mesenchymal stem cells
NBT: Nitro blue tetrazolium
NF-*κ*B: Nuclear factor kappa B
OMSCs: Oral mucosa mesenchymal stem cells
PC: Protein carbonyl
ROS: Reactive oxygen species
SC: Stem cell
SDS: Sodium dodecyl sulfate
SOD: Superoxide dismutase
TAC: Total antioxidant capacity
TBA: Thiobarbituric acid
TBI: Traumatic brain injury
TNF-*α*: Tumor necrosis factor-*α*
TPTZ: 2, 4, 6-tri-pyridyl-s-3, 6 triazine
Veh: Vehicle.
